# Emotional intelligence among medical students in Sweden – a questionnaire study

**DOI:** 10.1186/s12909-023-04570-0

**Published:** 2023-08-24

**Authors:** Aziz Bitar, Lava Amnelius, Emelie Kristoffersson, Jens Boman

**Affiliations:** Department of Clinical Science, Professional Development, Umeå University, Umeå, 901 87 Sweden

**Keywords:** Emotional intelligence (EI), Swedish medical students, Questionnaire

## Abstract

**Background:**

Emotional intelligence (EI), the ability to understand and regulate one’s and other’s emotions, has been linked to academic and clinical performance and stress management, making it an essential skill to develop during medical school. Nevertheless, uncertainty remains about the impact of medical education on EI, its association with sociodemographic factors, and the potential moderating role of gender. Therefore, this study aimed to explore levels of global EI among Swedish medical students based on their completed semesters while analyzing the potential moderator role of gender and identifying potential EI differences associated with age, gender, prior education, work experience, and previous experience working in a leadership position.

**Methods:**

The participants were medical students in semesters 1, 3, 5, 7, 9, and 11 at a Swedish University. Participants answered the self-report Trait Emotional Intelligence Questionnaire - Short Form (TEIQue-SF) and demographic questions. For each participant, the mean global trait EI was calculated (range 1–7), and differences were compared based on semesters and sociodemographic factors. In addition, we investigated the relationship between semester and EI scores with gender as a moderator.

**Results:**

Of the 663 invited medical students, 429 (65%) responded, including 269 women (62.7%), 157 men (36.6%), and 3 identifying as others (0.7%). The participants had a mean global trait EI score of 5.33. Final-year students demonstrated significantly higher global trait EI scores than first-year students, and gender did not have a moderating effect across semesters. Furthermore, students in the age group 25–29 years showed higher EI scores compared to those in the age group 21–24 years, while there were no significant differences in EI scores for older students (≥ 30 years) compared to other age groups. Higher EI scores were also positively associated with previous work-and leadership experiences. Gender and previous education did not significantly impact EI scores.

**Conclusions:**

Our findings suggest that higher EI scores are associated with semesters of medical education, age, and previous work and leadership experience. Future longitudinal studies are needed to identify factors that could improve EI among medical students to design curricular activities aimed at supporting the EI of the next generation of physicians.

## Background

In recent years, emotional intelligence (EI) has gained increased attention in medical education and research [1–3]. EI is often defined as the ability to perceive, express, understand, and manage emotions [1, 4, 5] and consists of the components: self-awareness, self-regulation, motivation, empathy, and social skills [6]. However, the exact definition of EI has been the subject of debate. The literature has three primary ways of conceptualizing and measuring EI [5, 7]. The ability model describes EI as a cognitive-emotional ability, like intelligence quotient (IQ), evaluated through performance-based questions that assess social skills, such as solving emotion-related problems. The trait model views EI as a trait, i.e., behavioral tendencies over time, assessed through self-evaluation. Finally, the mixed model considers EI as a combination of social skills, traits, and competencies, measured via questionnaires. Lastly, it should be kept in mind that the different ways of conceptualizing EI overlap and can co-exist [8]. In the present study, we used the trait EI model, due to its high validity in contexts characterized by ongoing stressors, such as medical education [5].

Whether EI is considered an ability or a trait, research has shown that EI underlies professionalism, communication skills, and other competencies essential for physicians [1, 9–11]. In medical settings, higher levels of EI have been associated with successful leadership, resulting in greater productivity and efficiency [7, 12–15]. High EI also predicts better academic performance [4, 16, 17] and is associated with lower stress and anxiety levels [18–20]. Some evidence also indicates that EI could protect against burnout [21, 22].

Given the high demands on quality and productivity in health care, coupled with the increasing prevalence of mental illness among medical students and physicians [23, 24], it is vital to equip future physicians with skills to cope with the demanding and stressful academic and clinical settings. Studies have suggested that improving EI among medical students and physicians could lead to better medical leadership, enhanced patient-physician relationships, and improved health outcomes [7, 25]. Thus, developing and incorporating educational interventions to enhance EI in medical students could have far-reaching benefits for the healthcare system.

Whether EI can be trained and modified in medical education has been debated – but if viewed as a behavioral trait it should be regarded as dynamic and thus possible to influence. One review study showed that medical students who undergo EI training demonstrated improvement [26]. However, it is important to note that the studies included in the review focused primarily on interventions for empathy rather than EI itself. While the interventions discussed may enhance empathy skills – which are indeed closely related to EI – the direct impact of these interventions on broader EI domains in medical students is less clear. Only one study revealed a borderline significant effect on EI after educational interventions [27].

To successfully design, test, and establish practical training in EI, it is essential to first consider factors that could influence trait EI, such as gender, age, and previous work experience. Many studies have explored the association between medical students’ sociodemographic factors and EI – according to a recent scoping review, though, the results are inconclusive [1]. Some studies show that women have higher EI than men [28, 29], while others have found no differences between women and men [30–34]. Furthermore, it is unclear whether these possible gender differences persist across different stages of medical education or whether they are influenced by the progression of semesters. Moreover, limited research has explored the potential moderating role of gender in trait EI, particularly in relation to completed years of medical studies. With regards to age, some studies have found higher age to be positively associated with EI [35, 36]. Other studies, in turn, found no explicit agreement between medical students’ EI, age [31, 37], and years of completed education [31, 34]. Likewise, some studies have shown that EI decreases over time [38], while others that it increases [29]. Furthermore, although many studies have explored the association between EI and different sociodemographic variables, few studies have investigated the importance of previous education, previous work experience [39], and leadership experiences. Thus, more research is needed on the association between medical students’ trait EI and factors such as age, gender, previous work experience, and leadership experiences.

The Swedish Higher Education Authority states that every student that has finished medical school should be able to show leadership and interprofessional cooperation, self-reflection, and empathy and provide a professional approach [40]. The Swedish Higher Education Authority does not explicitly mention EI in its national goals for medical education. However, their outlined skills and competencies align with EI components such as empathy and self-reflection. Still, to our knowledge, previous studies have yet to investigate EI in a Swedish medical education context.

Understanding factors associated with higher trait EI among medical students is important for designing effective educational interventions aimed at strengthening their EI. By identifying if gender moderates the relationship between semesters and EI it is also possible to tailor these interventions to ensure equal opportunities for EI development. Therefore, this study aimed to investigate levels of global trait EI among Swedish medical students based on their completed semesters, while analyzing the potential moderator role of gender and identifying potential EI differences associated with age, gender, prior education, work experience, and previous experience working in a leadership position. In addition, the goal was also to assess the need to develop educational interventions focused on enhancing EI among medical students to prepare them for their demanding profession as physicians.

## Methods

### Design

We chose a cross-sectional study design to explore EI among Swedish medical students, as no previous research has been conducted on this topic. We used a self-report questionnaire based on the trait EI model to measure EI since it could be administered to a large number of participants – and as described in the background: since the trait EI measure has high validity in contexts characterized by enduring stressors, such as medical education [5]. We included participants from various semesters, including first and final-year students, to assess whether there were any differences between students enrolled in different semesters.

### Setting

This study was performed at Umeå University Medical School where enrollment takes place twice a year. Students participating in this study followed a 5,5 year (11 semesters) undergraduate curriculum, including 3 years of clinical training. After graduation, students fulfill an 18-month internship granting them a medical license.

### Measures

The Swedish version of the Trait Emotional Intelligence Questionnaire-Short form (TEIQue-SF) [41, 42] was used to assess the students’ EI. This questionnaire has been previously validated and shown to have good reliability for global trait EI. The TEIQue-SF is a shorter version of the original Trait Emotional Intelligence Questionnaire (TEIQue).

The TEIQue-SF is a self-report questionnaire that measures trait emotional intelligence and consists of 30 items. Each item can be categorized into four broad factors: Emotionality, Sociability, Well-being, and Self-control reliability for global trait [43, 44]. The item: “I find it difficult to bond well even with those close to me”, for example, fall under the factor Sociability. Emotionality reflects the capacity to recognize and express emotions and use these abilities to uphold relationships. Self-control measures the capability to regulate one’s emotions, sustain impulse control, and manage stress. Sociability estimates skills in social interactions, e.g., in communication and networking. Well-being reflects a general sense of happiness and fulfillment.

Each item in the TEIQue-SF is scored on a 7-point Likert scale ranging from 1 (strongly disagree) to 7 (strongly agree). The mean score of all 30 items is calculated, providing a mean EI value, with a maximum score of 7 and a minimum score of 1. Of these items, 15 are inversely related to high EI and must be inverted before calculating the score. The mean EI value, also known as global trait EI, reflects overall emotional functioning and the perceived ability to interpret, process, and use information about one’s own emotions and those of others [41].

For this study, a two-section questionnaire was created using Microsoft Forms. The first section contained questions on sociodemographic information. Students’ semester, age group, and whether they had any previous university education, work experience, or experience of working in a leadership position were assessed using questions with predefined response options. The question of whether they had any previous work experience was followed up with an open-ended question asking them to specify for how long. Students’ gender was also assessed using an open-ended question. The second section of the questionnaire contained the TEIQue-SF items.

### Participants

The questionnaire was distributed to all medical students registered in semesters 1, 3, 5, 7, 9, and 11, a total of 663 students, through the study administrators for the respective semester. The number of students registered in the studied semesters was obtained from both the student register and study administrators.

### Data collection

During the Spring term of 2021, the questionnaire, together with information about the study, was distributed to the potential participants via e-mail. The information stated that participation was anonymous and voluntary. After the e-mails were sent out, the second author presented the study during one of the lectures for each semester, providing further information about the investigation. However, it was not possible to hold a presentation for semester seven. Following the initial e-mails and presentations, two reminders with a link to the questionnaire were sent to all participants via e-mail to encourage participation.

### Statistical analyses

From Microsoft Forms, the data were exported to Microsoft Excel and subsequently imported into the software IBM SPSS Statistics 28 for analysis. A global trait EI value was calculated for each participant, and a comparison was made between the semesters and the demographic groups.

All the TEIQue-SF items in the datasets were complete, and the sociodemographic variables had less than 1% of missing values for any variable. Four participants did not complete the question about previous work experience and one participant did not answer the question about age and were hence excluded from the respective analysis. Answers to the open-ended sociodemographic questions were reviewed and grouped into categories (see Table 1) to enable statistical analysis. Answers to the question about students’ gender were sorted into three categories; ‘woman’, ‘man’, and ‘other’. The length of previous work experience was grouped in two-year intervals.

**Table 1:** Demographic characteristics of the participants and mean global trait EI for all subgroups

	Number of participants (%)	Mean value of global trait EI (± SD)	95% CI for mean value of Global EI
Total	429 (100.0%)	5.33 ± 0.60	5.27–5.39
Semesters			
1 (first year)	109 (25.4%)	5.23 ± 0.62	5.11–5.34
3	78 (18.2%)	5.31 ± 0.61	5.17–5.44
5	60 (14.0%)	5.36 ± 0.58	5.21–5.50
7	68 (15.9%)	5.40 ± 0.57	5.26–5.53
9	58 (13.5%)	5.25 ± 0.63	5.08–5.40
11 (final-year)	56 (13.1%)	5.53 ± 0.50	5.40–5.67
Age			
≤ 19	33 (7.7%)	5.24 ± 0.65	5.00–5.47
20–24	208 (48.5%)	5.25 ± 0.61	5.17–5.33
25–29	122 (28.5%)	5.45 ± 0.50	5.36–5.54
≥ 30	65 (15.2%)	5.42 ± 0.65	5.28–5.39
Gender			
Women	269 (62.7%)	5.34 ± 0.57	5.27–5.41
Men	157 (36.6%)	5.32 ± 0.62	5.23–5.42
Other	3 (0.7%)	4.66 ± 1.20	1.69–7.62
Previous education*			
None	219 (51.0%)	5.28 ± 0.58	5.21–5.36
1–3 yrs*	164 (38.3%)	5.34 ± 0.61	5.25–5.44
> 3 yrs*	46 (10.7%)	5.50 ± 0.61	5.32–5.69
Previous work experience			
None	115 (26.8%)	5.21 ± 0.71	5.08–5.34
0–2 yrs	183 (42.7%)	5.31 ± 055	5.23–5.39
2–4 yrs	31 (7.2%)	5.44 ± 0.54	5.23–5.63
4–6 yrs	52 (12.1%)	5.47 ± 0.49	5.36–5.61
> 6 yrs	44 (10.3%)	5.33 ± 0.59	5.30–5.64
Previous leadership position			
No	358 (83.4%)	5.29 ± 0.60	5.23–5.36
Yes	71 (16.6%)	5.52 ± 0.54	5.39–5.66

*=education on a postsecondary level

Since normality assumptions were met, one-way ANOVAs (more than two groups) or independent t-tests were used to analyze the relationship between global trait EI scores and groups categorized: semesters, ages, gender, previous education, previous work experience, and previous experience in a leadership position. The groups were used as the independent variable, and the EI scores as the dependent variable. Whenever a one-way ANOVA was significant, we performed a follow-up analysis.

When comparing the relation between semester groups and EI, post-hoc analyses were performed using Dunnett’s tests, with first-year medical students as the reference group.

To analyze the moderating effect of gender on the association between semesters and EI, a series of hierarchical regression analyses were performed. In the first step, gender was included as a predictor variable. The second step involved the inclusion of the semester groups. In the third step, a multiplicative term semester × gender was added.

For comparing the length of previous work experience and EI, a significant one-way ANOVA was followed by Dunnett t-tests that treated one group as a control (i.e., students with no prior work experience) against all other groups.

A one-way ANOVA was also used to compare the global trait EI scores across age groups. Due to small sample sizes in higher age groups, participants were recoded into a new group labeled > 30 years for statical purposes. Post-hoc pairwise analyses were conducted using the Tukey HSD tests to determine between which groups there were significant differences.

The independent sample t-test was employed to compare the mean global trait EI values between men and women. Three participants who defined themselves as ‘other’ were excluded from the analysis due to the small sample size. Additionally, the same analysis was performed for those with and without experience of being in a leadership position.

### Ethical considerations

The participants in the study received information about the study, and they provided their informed consent to participate by returning their completed questionnaires. The questionnaire was anonymous and voluntary, and no personal information was gathered. Therefore, the study was considered exempt from requirements for approval by the Swedish Ethical Review Authority under The Swedish Act (2003:460) concerning the ethical review of research involving humans.

## Results

### Demographic characteristics and global trait emotional intelligence

Of the 667 invited medical students, 429 responded, resulting in a response rate of 64%. Of the participants, 62.7% were women, 36.6% were men, and 0.7% identified as others. The largest age group was 20–24 years, comprising 48% of the participants. 73% of the students reported having prior working experience, and 17% reported having previous leadership experience. Furthermore, 51% of participants reported having a previous university education. The overall mean value for global trait EI was 5.33 (95% CI: 5.27–5.39).

The demographic characteristics and mean global trait EI scores for subgroups are presented in Table 1.

### Relationship between semesters and global trait emotional intelligence

A one-way ANOVA showed a significant difference in mean global trait EI between semester groups (F(5, 423) = 2.39, p = .037). Post hoc analyses indicated that only final-year students had a significantly higher mean global trait EI score than first-year students (p = .009), indicating a medium effect size (Cohen’s d of 0.52).

The comparison of mean global trait EI scores between first-year students and those in other semesters is shown in Table 2.

**Table 2:** Comparison of mean global trait EI between first-year students and students in other semesters

Attending semester	Mean Difference	Std. Error		Effect Size (Cohen’s d)		
3	0.08	0.09			-	
5	0.13	0.10			-	
7	0.16	0.09			-	
9	0.02	0.10			-	
11	0.30*	0.10			0.52	

*Dunnett t-test treat one group as a control (Semester 1, i.e., first-year students) against which all other groups are compared. The comparisons are based on a 7-point Likert scale*

*p = < 0.05

### Comparison of global trait emotional intelligence and demographic characteristics

A one-way ANOVA revealed a significant overall difference in EI scores across age groups (F(3, 424) = 3.81, p = .010). Post hoc analyses indicated that only participants in the age group 25–29 had higher global trait EI scores than those in the age group 20–24 (p = .016).

A one-way ANOVA showed a significant difference in mean global trait EI between students with previous work experience and those without (F(4, 420) = 2.84, p = .024). Post hoc analyses indicated that students with prior work experience of 4–6 years had a significantly higher mean global trait EI score than students without previous work experience (p = .032).

An independent t-test showed that students with previous experience working in a leadership position had significantly higher global trait EI scores than those without prior experience in a leadership position (t(427) = -2.89, p = .004).

An independent t-test and a one-way ANOVA, respectively, showed that neither gender (t(424) = 0.30, p = .761) nor previous university education (F(2, 426) = 2.69, p = .069) had a significant impact on global trait EI scores.

### Moderating effect of gender on the relationship between semesters and emotional intelligence

The regression analyses revealed no significant moderating effects of gender on the relationship between semesters and EI scores (-0.05, p = .548).

The results from the regression analysis are presented in Table 3.

**Table 3:** Moderating effect of gender on the relationship between semesters and emotional intelligence

	*R* ^*2*^	*F*		*β*		
Step 1	0.00	0.90				
Gender					− 0.02	
Step 2	0.01	2.70				
Gender					− 0.02	
Semester					0.11*	
Step 3	0.1	1.92				
Gender					0.02	
Semester					0.13*	
Semester x gender					− 0.05	

* p = < 0.05

## Discussion

In this study, we investigated global trait EI scores among undergraduate medical students in Sweden based on their completed semesters of education while analyzing the potential moderator role of gender and exploring potential EI differences associated with sociodemographic factors. We found that the participants had a mean global trait EI score of 5.33. Final-year students had significantly higher global trait EI scores than first-year students, but our study showed no moderation role of gender on the association between EI scores and semester. Furthermore, age, previous work experience, and previous experience in a leadership position were positively associated with higher EI scores. In contrast, gender and previous university education did not significantly impact EI. These results provide insights into factors associated with higher EI scores.

### Factors affecting emotional intelligence

This is the first Swedish study exploring EI among medical students. Our study showed that the participants had a mean global trait EI score of 5.33, which is higher than in studies conducted on medical students in other countries [2, 33, 45–47]. Previous research suggesting that EI rises with increasing age could explain our findings since Swedish medical students generally enter their undergraduate studies at an older age (mean age of 24 years) than medical students in other countries [48]. Our study also found a significant difference in EI between age groups supporting previous studies [35, 36]. Interestingly, our analysis showed the age group 25–29 had significantly higher EI scores than the age group 20–24, while no significant difference was observed for the age group ≥ 30 when compared to other age groups. This suggests that age up to a certain level can influence students’ EI, but additional factors other than age may be required to continue developing this ability beyond that level. For example, in this study, the majority of students in the age group 25–29 are final-year students, so their higher EI scores could be influenced by factors related to being in the final year, such as increased clinical exposure, rather than age itself.

Our results also showed a significant difference in mean global trait EI scores between first-year and final-year medical students with final-year students scoring higher, with a medium effect size (Cohen’s d = 0.52). This is an interesting finding since one previous, longitudinal, study has found significant improvement in EI over a five-year period among medical students suggesting that EI improve as students progress through their education [29]. Our study, however, is cross-sectional, so we cannot draw any such conclusions on causality. Thus, more longitudinal studies are needed to determine whether EI increases as a result of medical training – and if so, which aspects of the training contribute to this increase. Such studies could also provide clues into how future interventions can be designed to enhance students’ EI.

Also, our study showed that medical students with previous work experience had significantly higher EI scores than those without such experience, a finding consistent with previous research [39]. Our study, however, adds to earlier studies by demonstrating that it may take at least four to six years of work experience to significantly impact EI. One possible explanation is that work experience may expose students to diverse perspectives and situations, providing them with opportunities and situations to develop better communication and leadership skills, which can further facilitate their ability to understand and manage their own and others’ emotions. The awareness that relatively long work experience is required to strengthen EI could be important to consider when evaluating different merits in admission to medical school since it is imperative that the most suitable students are admitted. However, it is important to note that long work experience per see is no guarantee of high EI. Even more important than taking into account the length of any previous work experience would be to consider students’ actual EI in the admission process. However, previous studies have pointed to the difficulties in such a procedure in terms of the risk of accepting or rejecting the wrong students [49].

Furthermore, students with previous leadership experience also displayed higher EI scores compared to those without such experience. This is important since previous research suggests a strong link between EI and effective leadership [7, 12–15]. Additional research could explore whether leadership experience promotes EI development or whether individuals with higher EI are more likely to be selected for leadership roles.

In this study, we found no significant gender differences in trait EI among medical students, consistent with some previous studies [30–34] – nor did our moderation analysis indicate that gender moderated the relationship between semester and global EI scores. The finding that, in contrast to studies from several other countries, there were no differences between men and women in levels of EI could be due to Sweden’s strong position in terms of gender equality [50] which means that they have the same rights, obligations, and opportunities to develop their EI. Thus, our findings also suggest that all medical students, regardless of gender, could benefit equally from interventions directed at developing their EI skills.

Swedish universities, like those in many other countries, have intended learning outcomes for teaching empathy, self-awareness, and stress management – all of which could be seen as aspects of EI whether or not the term EI per see is mentioned in the curriculum. The participants in our study had relatively high EI. However, the medical profession is demanding, and there is certainly room for improving EI further. Furthermore, previous studies have shown an increasing prevalence of stress and mental illness among medical students and physicians [21, 23]. This is worrisome and could perhaps be countered by introducing more training on EI since aspects related to EI have been shown to be essential for mental health [51]. According to previous studies enhancing EI in future physicians could equip them, not only to handle the stressors of working life [18–21] but also to help them reach their full academic and clinical potential [4, 16, 17]. Thus, future longitudinal studies are needed to identify factors that could improve EI among medical students; e.g., to design curricular activities aimed at supporting the EI of the next generation of physicians.

### Methodological considerations, strengths, and limitations

In this cross-sectional study, we used the short-form TEIQue-SF questionnaire to explore self-reported EI among medical students over several semesters. Our study design allowed us to capture a snapshot of EI among a large group of medical students (N = 429) and examine relationships between EI and several sociodemographic factors. The response rate was high (64%). Furthermore, the study population reflected the age distribution and the distribution of men and women at Umeå medical school. According to previous studies, women and men comprise approximately 60% and 40%, respectively, of the student body, and the mean age of first-year and last-year medical students is 22 versus 27 for women, and 23 versus 28 for men, respectively [52, 53].

However, the study has several limitations that must be considered. First, the cross-sectional design means that we cannot determine cause and effect, such as the effect of semesters in medical school. Thus, future studies should adopt a longitudinal design and follow a group of medical students, assessing their EI over time. Second, as mentioned above, the factors analyzed in this study can confound since age likely correlates with both semesters, work experience, and leadership experience. Third, using a self-report questionnaire like the TEIQue-SF to measure EI always carries the risk of social-desirability bias. However, the questionnaire was anonymous and for research purposes only, thus minimizing the risk of such response bias [54]. Fourth, the study was conducted during the COVID-19 pandemic, which could have affected the outcomes and replicability of the results – partly because studies have indicated that the pandemic may have had a negative impact on mental health [55, 56], which could affect students’ responses. Lastly, the study sample, which only included participants from one medical school, constitutes a limitation regarding the generalizability of our findings. Future studies should therefore include students from multiple universities to improve the external validity of the results.

Despite the limitations described above, our study adds to the knowledge of EI among medical students and generates insights that can be used in future research and interventions to improve medical students’ EI.

## Conclusions

In this study, we explored levels of global trait EI among Swedish medical students based on their completed semesters while analyzing the potential moderator role of gender and explored potential differences in EI associated with age, gender, previous education, work experience, and previous experience working in a leadership position.

The results showed that the participants had a mean global trait EI score of 5.33. Final-year students demonstrated higher global trait EI scores than first-year students and gender did not have a moderating effect across semesters. In addition, our results showed a higher EI among students in age groups 25–29 years compared to age-group 21–24 years, suggesting that age up to a certain level can influence students’ EI. Furthermore, students with previous work experience or experience in a leadership position showed significantly higher EI than students without such experiences. Comparisons between men and women did not reveal significant differences in EI scores.

The medical profession is demanding and higher EI could help students to handle the stressors of working life and to reach their full academic and clinical potential. There is a need for future longitudinal studies to identify factors that could improve EI among medical students to design curricular activities aimed at supporting the EI of the next generation of physicians.

## Data Availability

The datasets generated during this study are available from the corresponding author on reasonable request.

## List of abbreviations

EI: Emotional intelligence
TEIQue: Trait Emotional Intelligence Questionnaire
TEIQue-SF: Trait Emotional Intelligence Questionnaire – Short Form
Global EI: Global Emotional Intelligence, total score on TEIQue-SF for each participant
